# Manifestation of a Vestibular Schwannoma in a Patient With PHACE Syndrome

**DOI:** 10.7759/cureus.78946

**Published:** 2025-02-13

**Authors:** Alejandra Viera Plasencia, Laura Amador, Jefry Biehler, Julie Kantor

**Affiliations:** 1 Department of Pediatrics, Herbert Wertheim College of Medicine Florida International University, Miami, USA; 2 Department of Pediatrics, Nicklaus Children's Hospital, Miami, USA

**Keywords:** cardiovascular anomalies, congenital, hemangioma, neurocutaneous disorders, neurodevelopmental, ocular anomalies, phace syndrome, vestibular shwanomma

## Abstract

This case study reports a 6-week-old female with PHACE syndrome and a vestibular schwannoma, an exceedingly rare combination not previously documented in medical literature. PHACE syndrome, characterized by posterior fossa malformations, large facial hemangiomas, and arterial anomalies, manifested in the patient with an enlarging facial hemangioma obstructing her left eye. Imaging studies revealed left cerebellar hypoplasia, absent right vertebral artery, absent left A1 portion of the anterior cerebral artery, and a suspected acoustic neuroma in the left cerebellopontine angle. Additionally, a possible thyroid hemangioma and a small hepatic hemangioma were observed.

The presence of a vestibular neuroma in PHACE syndrome, typically associated with Neurofibromatosis Type 2 or sporadic cases, poses a unique challenge. This unusual presentation raises critical considerations for differential diagnosis, given that vestibular schwannomas are not a known feature of PHACE syndrome. The coexistence of vascular anomalies and a vestibular schwannoma complicates imaging interpretation and may influence diagnostics and treatment choices. This case underscores the complexity and rarity of such presentations, highlighting the need for further research to understand the etiology and treatment of vestibular schwannomas in PHACE syndrome patients, given the absence of prior documented cases of this combination.

## Introduction

PHACE syndrome is a complex neurocutaneous disorder that affects blood vessels and tissues in different parts of the body, including the brain, heart, and eyes. It is named after its key features: (P) posterior fossa malformations (abnormalities in the back of the brain), (H) large facial hemangiomas (birthmarks made of blood vessels), (A) arteriovenous anomalies (abnormal blood vessel formation), (C) cardiovascular anomalies, and (E) eye anomalies. Additionally, sternal clefting is sometimes present [1].

While PHACE syndrome is sporadic and non-heritable in a clear Mendelian pattern, its underlying cause remains unclear [2]. Given its female prominence and prenatal male lethality, it is believed to be caused by mutations in the X chromosome that affect the process of vasculogenesis. Given that the malformations occur on the same side, it suggests an abnormality in embryonic development [2].

The literature is very limited regarding the relationship between the presence of acoustic neuromas, also known as vestibular schwannoma (VS), and PHACE syndrome. Vestibular schwannoma is a benign Schwann-cell tumor of the vestibulocochlear nerve [3]. This tumor originates from mutations in the NF2 tumor suppressor gene present on chromosome 22 [3]. In patients with sporadic vestibular schwannoma, the tumor is usually unilateral and present in the absence of other brain tumors. In contrast, in patients with Neurofibromatosis type 2 (NF2) which is an autosomal-dominant syndrome with multiple neoplasms, the vestibular schwannoma arises bilaterally and may be present with other brain tumors [4]. 

The purpose of this case study is to document and analyze the unusual clinical presentation of a patient with a vestibular neuroma in a child with PHACE syndrome, a combination not previously reported. This case report is significant because PHACE syndrome is extremely rare, being present in less than 1 in 10,000 live births [5], and the clinical presentation is complex. It also aims to expand the understanding of PHACE syndrome, especially its clinical manifestations. By comparing the clinical presentation and results of this study with the available literature, this report aims to expand the knowledge and understanding of its clinical implications and potential diagnostic challenges. This report also discusses a rare additional clinical condition not previously reported in association with PHACE syndrome, a vestibular neuroma.

## Case presentation

**Figure 1:**
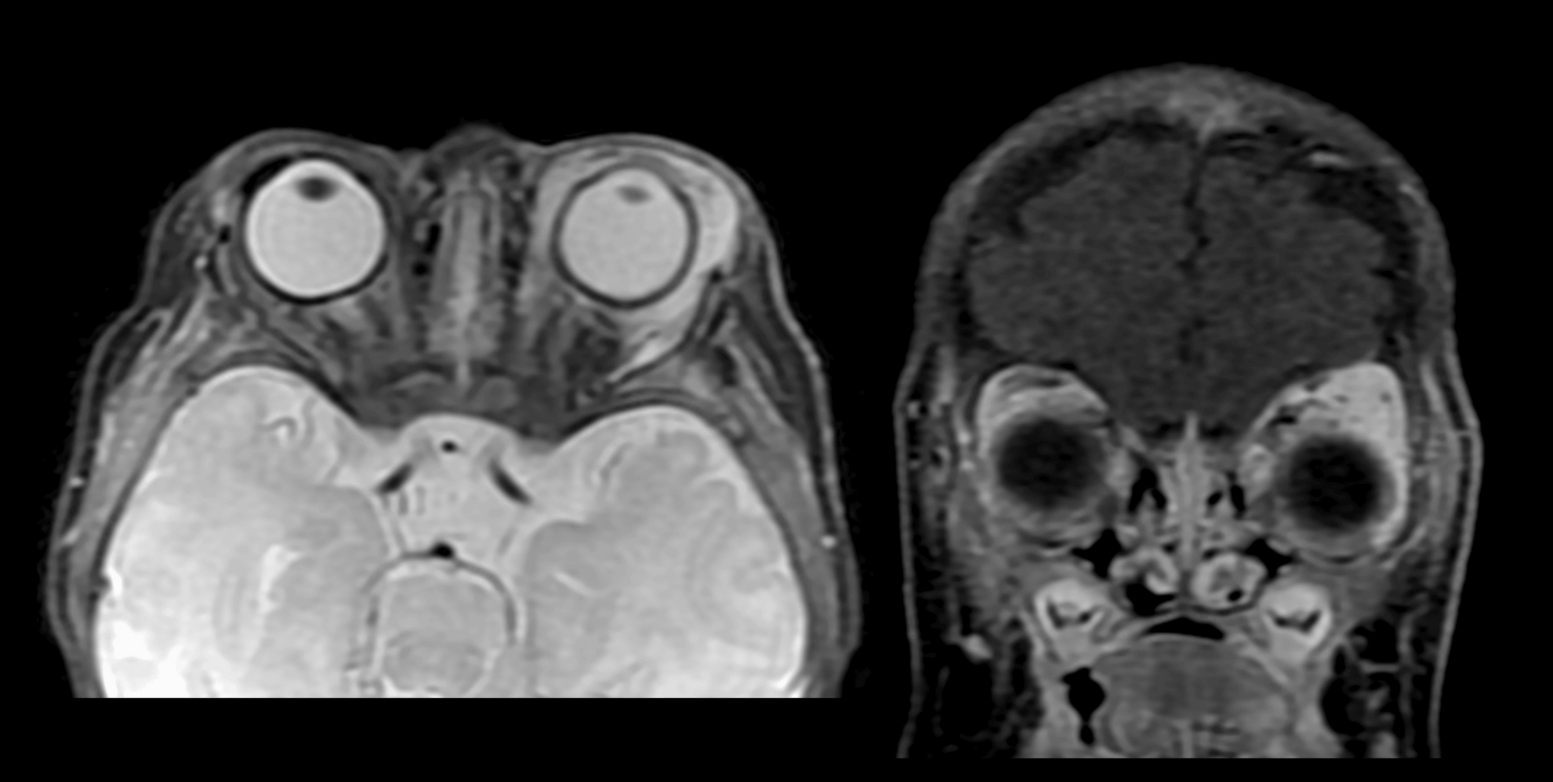
Facial MRI showed a left orbital hemangioma with pre- and post-septal extension. The lesion caused significant eyelid swelling, leading to restricted mobility and partial obstruction of vision.

A 6-week-old female, with a medical history of a failed birth hearing screen, presented to the Emergency Department with an enlarging facial mark extending over the left side of the forehead, eyelid, and cheek. The patient was experiencing restricted eyelid mobility, due to significant swelling of the birthmark. An MRI of the face showed signal abnormality in the left orbit with pre- and post-septal extension consistent with infantile hemangioma, without intraconal extension (Figure 1). The left globe was intact, and the right orbit was unremarkable. Additionally, a signal abnormality was observed in the left thyroid region with minimal enhancement, suggestive of a small hemangioma. Cervical lymph nodes showed a dominant right jugular digastric lymph node and bilateral spinal accessory lymph nodes, while the parotid and submandibular glands appeared unremarkable.

**Figure 2:**
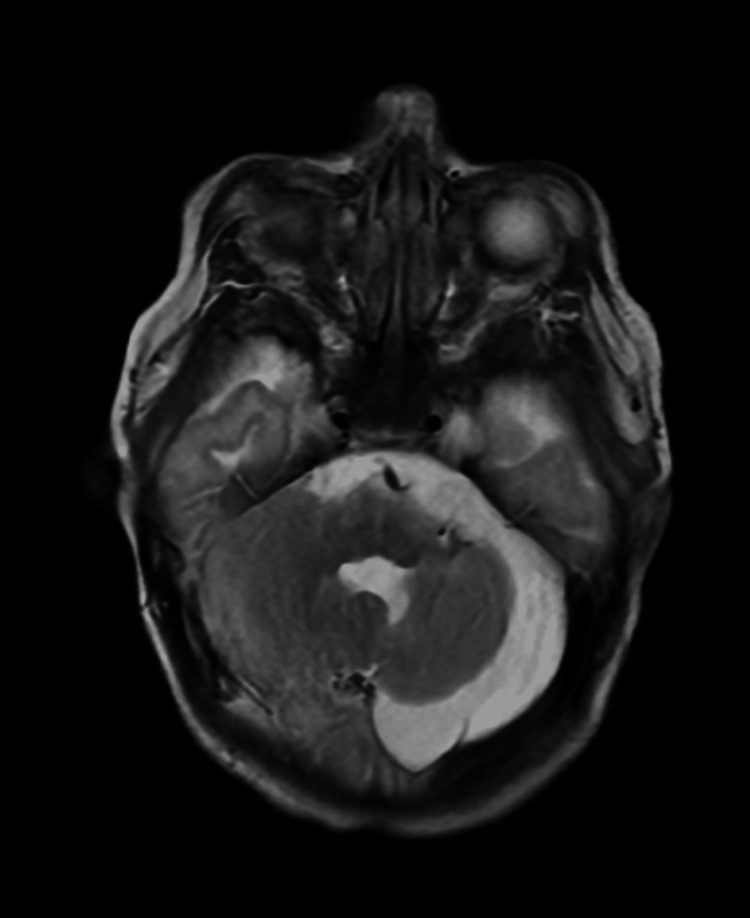
Brain MRI with contrast showed left cerebellar hypoplasia and an enlarged infratentorial subdural space. Cerebellar hypoplasia may contribute to potential developmental delays, balance difficulties, and neurological deficits in PHACE syndrome.

**Figure 3:**
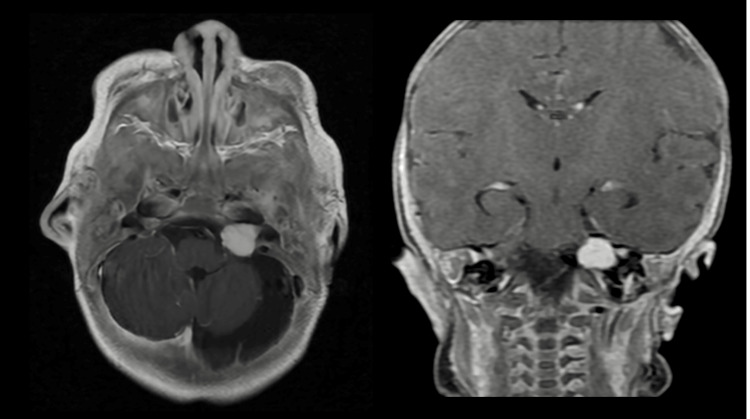
Brain MRI with contrast showed a 1.1 cm contrast-enhancing mass in the left cerebellopontine angle, suggestive of a vestibular schwannoma. This finding correlates with the patient’s failed newborn hearing screening, raising concerns about congenital auditory dysfunction unrelated to the typical vascular anomalies seen in PHACE syndrome.

Consequently, an MRI of the brain with and without contrast showed left cerebellar hemisphere hypoplasia, enlargement of the left fourth ventricle lateral recess, and an increased infratentorial subdural space extending along the left cerebral hemisphere (Figure 2). A contrast-enhancing mass measuring 1.1 cm in anteroposterior and transverse dimensions and 1 cm in craniocaudal dimension was identified in the left cerebellar pontine cistern along the left internal auditory canal, suggesting an acoustic neuroma (Figure 3). The abnormal signal was noted in the mastoid air cells. The right vertebral artery was absent, and the left A1 portion of the anterior cerebral artery was missing. These findings are indicative of PHACE syndrome, including left cerebellar hypoplasia, arterial abnormalities, and left orbital hemangioma. The critical findings of the patient's imaging studies are summarized in Table 1. An abdominal ultrasound revealed a hypervascular lesion, slightly hypoechoic and triangular, measuring approximately 1.0 x 0.7 x 1.5 cm in the left hepatic lobe, likely representing a hemangioma. The pancreas showed no abnormalities. The patient was admitted for management of the facial hemangioma and initiated on oral propranolol. The dose was titrated over the course of three days. The swelling on the left eye greatly improved, allowing the patient again to open her eye.

**Table 1:** Summary of imaging studies and main findings

Imaging Studies	Findings
MRI of the face	Facial hemangioma, possible thyroid hemangioma.
MRI of the brain	Left cerebellar hemisphere hypoplasia, possible left acoustic neuroma, absent right vertebral artery, absent left A1 portion of the anterior cerebral artery.
Abdominal ultrasound	Possible left hepatic lobe hemangioma.

## Discussion

PHACE Syndrome is a genetic disorder that involves different organ systems and is associated with infantile segmental hemangioma [6,7]. The most common features are abnormal development of the posterior fossa, especially the cerebellum, multiple infantile hemangiomas, vascular abnormalities such as aortic anomalies, and eye anomalies. Posterior fossa malformations are anomalies of the part of the skull that contains the cerebellum and brainstem, which control balance and coordination [7]. The incidence of this syndrome is very low and can lead to life-threatening complications. 

The American Academy of Pediatrics provides a series of criteria to qualify this genetic condition as Definite PHACE Syndrome or Possible PHACE Syndrome [8]. The criteria for Definite PHACE Syndrome include the presence of one segmental hemangioma or hemangioma greater than 5 cm on the face or the scalp and either one major criterion from any organ system or two minor criteria from any organ system [8]. The criteria for Possible PHACE Syndrome include the presence of a hemangioma greater than 5 cm on the face or the scalp and one minor criterion from any organ system [8].

According to the results of the imaging studies performed on this patient, the patient meets the criteria for Definite PHACE Syndrome. The patient has a facial hemangioma that extends over the left side of her forehead, eyelid, and cheek. Additionally, the patient meets many of the major criteria from the cerebrovascular organ system, such as absent right vertebral artery and absent left A1 portion of the anterior cerebral artery (absence or moderate to severe hypoplasia of large cerebral arteries). The patient also meets the major criteria for structural brain anomalies (left cerebellar hemisphere hypoplasia) which is a well-documented posterior fossa anomaly in PHACE syndrome. The patient also has suspected hemangiomas on the thyroid gland and the liver, although these findings are not part of the classification criteria. Further studies are needed to establish whether visceral hemangiomas should be considered more routinely in PHACE syndrome patients and if their presence has prognostic or therapeutic implications.

This patient also has an acoustic neuroma, which is not a recognized component of the PHACE syndrome criteria. While vestibular schwannomas are typically associated with Neurofibromatosis Type 2, their presence in a patient with PHACE syndrome raises the question of a potential link between neural crest-derived tumors and vascular anomalies. There have been recent reports of patients with PHACE syndrome presenting with hearing loss. It was described as the case of a 4-year-old female with PHACE syndrome who presented with hearing loss [9]. An otoscopic examination of the patient showed a narrowed external auditory canal and an angiomatous lesion. In this case, a CT scan of the ears and mastoid revealed an angiomatous lesion in the middle ear. It was felt that this patient had conductive hearing loss secondary to an angiomatous lesion obstructing the middle ear [9]. Another study investigating hearing loss in PHACE syndrome patients revealed that while some patients experience conductive hearing loss, other patients experience sensorineural hearing loss. In the study, the laterality of the hearing impairment was ipsilateral to the location of the facial hemangioma. The conclusion was based on imaging studies and audiology exams. The etiology of the hearing loss in these patients was due to hemangiomas in the internal auditory canal that compressed the vestibulocochlear nerve [9]. 

However, unlike previously documented cases of hearing loss in PHACE syndrome caused by vascular lesions, this patient's hearing impairment appears to be caused by the presence of a congenital vestibular schwannoma. Given that both conditions involve abnormalities in embryonic development, one hypothesis is that vascular disruptions in PHACE syndrome may influence Schwann cell proliferation or migration. This distinction is critical because it broadens the spectrum of possible auditory manifestations in PHACE syndrome, suggesting that some cases may not be solely due to vascular abnormalities. This suggests an alternative mechanism of auditory dysfunction in patients with PHACE syndrome, which expands the differential diagnosis beyond hematoma-related hearing loss. 

Despite extensive evidence relating patients with PHACE syndrome and the presence of hearing loss, these are attributed to hematological anomalies rather than to acoustic neuromas as seen in this patient. According to the literature, sporadic vestibular schwannoma is very rare in the pediatric population and usually occurs in adults over 50 [10]. In the pediatric population, they are often linked to Neurofibromatosis type 2. However, this patient does not have NF2, making this an unusual and significant finding. A study evaluated patients presenting with unilateral VS with no clinical evidence of Neurofibromatosis type 2, similar to the findings in this patient. These patients were between 11 and 18 years old. The tumors were solid, and some had varying characteristics such as hypervascularity, meatal expansion, and secondary hydrocephalus [10]. It is important to point out that these tumors were sporadic, and the schwannoma present in this patient seems to be congenital given that she had a failed hearing screening at birth. 

The presence of VS in a patient with PHACE syndrome expands the spectrum of auditory complications and has important clinical implications. While hearing loss in PHACE syndrome is typically due to hemangiomas, this case suggests a potential risk for primary neoplasms affecting the vestibulocochlear nerve, requiring a different treatment approach. Unlike hemangiomas, which respond to propranolol, vestibular schwannomas require different treatments. Propranolol, the standard treatment for hemangiomas, would not be effective in this case, highlighting the importance of early audiological screening and neuroimaging in patients with unexpected hearing deficits.

After a review of the available literature on both PHACE syndrome and congenital schwannoma, no cases of coexisting illness were found. This unprecedented finding suggests a potential association that warrants further investigation. Understanding this relationship provides insight into the underlying genetic mechanism linking vascular anomalies to neural tumors, guiding future research. If future studies confirm a connection between PHACE syndrome and neural crest-derived tumors, this could redefine our approach to screening these patients. 

It is important for providers caring for patients with Definite PHACE Syndrome, to coordinate specialist care between genetics, dermatology, neurology, radiology, and otolaryngology. Regular monitoring for potential complications of hearing loss or neurological deficits is important. Given the implications of this case, future research should investigate the prevalence of neural crest-derived tumors in PHACE patients to determine whether a broader surveillance strategy is needed. Future research should focus on the relationship between PHACE syndrome and conditions like VS, with the goal of improving early detection and management.

## Conclusions

In conclusion, this case report documents a rare presentation of PHACE syndrome complicated by the presence of a vestibular schwannoma, an occurrence not previously described in the literature. The comprehensive assessment of this patient's clinical manifestations, including facial hemangioma, cerebrovascular abnormalities, and structural brain anomalies, confirms the diagnosis of PHACE Syndrome. The incidental discovery of a vestibular schwannoma contributes to our recognition of potential complications associated with this syndrome. While the literature has linked PHACE syndrome with hearing loss secondary to hemangiomas obstructing the middle ear, the presence of an acoustic neuroma in our patient suggests an additional distinct etiology. This report highlights the importance of considering rare manifestations in patients with genetic syndromes and emphasizes the need for further research to elucidate the underlying mechanisms and optimal management strategies for impacted patients. By contributing to the expanding body of knowledge on PHACE syndrome, this report aims to facilitate improved diagnostic accuracy and patient care in clinical settings.
